# Sem imparcialidade: reflexões antropológicas sobre silenciamentos, denegações e presenças-ausentes do racismo na saúde

**DOI:** 10.1590/0102-311XPT091826

**Published:** 2026-08-10

**Authors:** Washington Castilhos

**Affiliations:** 1 Fundação Oswaldo Cruz, Fiocruz, Rio de Janeiro, Brasil.

Há exatos dez anos, Jurema Werneck assinalou, em artigo ^1^, que nem mesmo o reconhecimento do racismo como um dos fatores centrais na produção de iniquidades em saúde fora suficiente para que a saúde da população negra - como campo de pesquisa e ação - ocupasse lugar relevante nas Ciências da Saúde. De fato, passada uma década, o tema segue sub-abordado no campo referido por ela, em que o racismo é inclusive frequentemente denegado, evocando aqui o conceito de pacto denegativo, a aliança organizadora de vínculos que possibilita que grupos se organizem em relação àquilo que é tolerável e aceitável para seus membros ^2^, um mecanismo de defesa social e psicológica muito comum no Brasil, sustentado pela crença no mito da democracia racial brasileira.

Se por um lado o campo da Saúde ainda não enfrenta o debate sobre o racismo em saúde, por outro, na Antropologia o tema tornou-se um campo de pesquisa em expansão nos últimos anos, frequentemente interseccionando com a Antropologia da Saúde. O livro *Racismo e Saúde: Perspectivas Antropológicas Contemporâneas* ^3^, organizado por Rosana Castro & Ana Cláudia Rodrigues, resulta desse movimento.

Refletindo uma mudança contemporânea observada na Antropologia em relação ao perfil docente e discente, à agência e autoria negras e à visibilidade de suas contribuições - como informam as organizadoras -, as reflexões reunidas na coletânea, de perspectiva anticolonial e antirracista, são todas produzidas por estudantes negras e negros com base em suas pesquisas de mestrado e doutorado, fundamentadas sobretudo na epistemologia feminista negra.

O foco em perspectivas mais contemporâneas estimula a atualização de referenciais bibliográficos, tão necessária nos debates que os tempos atuais instigam. Além desse traço, também chama a atenção na obra o modo como as autoras e os autores dos nove estudos acionam múltiplas vozes em suas análises, valendo-se dos distintos lugares que ocupam como estudantes, antropólogas e antropólogos, ativistas, e mulheres e homens negros, marcadores indissociáveis nas interpretações realizadas, todas elas permeadas pela interseccionalidade de raça, gênero e classe, entre outras.

Ao apostar na perspectiva do “Eu sobre o Outro” para a ótica do “Nós sobre Nós”, o livro aponta uma importante inflexão: abandona a perspectiva em que o Eu - sujeito universal branco - define e estuda o não branco como o Outro - o “não-ser” ^4^ - para adotar uma perspectiva situada onde sujeitos racializados e subalternizados constroem saberes baseando-se em suas próprias vivências e/ou de sua comunidade.

Os estudos antropológicos podem funcionar - e frequentemente funcionam - como instrumentos de denúncia. A Antropologia, por meio de métodos como a Etnografia, permite documentar violações de direitos e desigualdades raciais como as descritas no livro, as quais são muitas vezes invisibilizadas ou naturalizadas. Nesse sentido, todas as reflexões da coletânea indagam o racismo de forma situada, posicionada e engajada, sustentando compromissos éticos e políticos de denunciar negligências, silenciamentos e denegações.

“*Você não encontrará neste texto imparcialidade, e sim uma etnografia feminista encarnada e situada*” (p. 75), informa uma das autoras logo no início de seu texto, em que entrelaça a escrevivência e a autoetnografia para relatar situações de negligência e maus-tratos sofridos pela tia, primas e irmã nos serviços de saúde de municípios da Região Metropolitana de Manaus, no Amazonas, que resultaram, inclusive, na morte da irmã, vítima fatal de procedimentos médicos mal realizados durante o parto.

Os compromissos ético-políticos assumidos pelas autoras e autores em suas escritas se expressam de vários modos nas páginas do livro, seja questionando a negligência por trás dos casos de mortalidade materna ou na atenção às mulheres negras com traço falciforme, seja indagando o sistema de morte silenciosa que envolve as pessoas negras no contexto da aids ou inquirindo a denegação do racismo nos documentos que orientam a formação de graduandos em Psicologia de uma universidade.

Esses efeitos do racismo nos roteiros de algumas doenças e na formação médica geraram, nos últimos anos, a produção de engajamentos antirracistas, que colocam em perspectiva as especificidades da Lei de Cotas, da Política Nacional de Saúde Integral da População Negra e de práticas coletivas e individuais.

Tais questões são discutidas na segunda parte da coletânea, iniciada por um estudo que discute o histórico mutualismo entre medicina e branquitude e o efeito das cotas raciais na propositadamente embranquecida Faculdade de Medicina da Universidade Federal de Minas Gerais (UFMG), onde até o fim do século passado a presença negra só se materializava nos “corpos não reclamados”, usados como objetos de estudo nas aulas de anatomia.

Digo “propositadamente embranquecida” porque na medida que o texto avança, detalhando a forma como essa exclusão foi construída historicamente na base sólida do pacto narcísico da branquitude ^5^, vamos percebendo como acontecem as dinâmicas de produção social do racismo institucional, favorecidas por contextos e estruturas sociais, políticas e econômicas que organizam as relações de poder. Trazendo a discussão para os dias atuais, o autor analisa como o ingresso de estudantes negras e negros naquela Faculdade (com a implementação das cotas raciais) desestabilizou o pacto narcísico e possibilitou a criação de um coletivo negro atuante, frente à persistente desproporção racial.

A leitura dos textos da coletânea inevitavelmente desperta lembranças em pessoas racializadas. Movido por uma dessas memórias, compartilho um pequeno relato de experiência: como jornalista de ciência e saúde, lembro do meu grande desconforto quando, na minha primeira cobertura de um congresso médico, por acaso realizado em Belo Horizonte, deparei-me com uma demarcada separação racial na festa que oficialmente abria o evento: de um lado, a categoria médica, toda branca, em seus vestidos longos e *black ties*; de outro, garçons e garçonetes, todos negros, que circulavam com suas bandejas pelo salão. O *apartheid* colonialista daquela noite nunca mais me saiu da cabeça. A leitura desse artigo em questão me fez enxergar com clareza - passados todos esses anos - que o que se passava naquela noite era a operacionalidade do ideário estético que atribui valor à categoria médica no imaginário social. Para ser médico, tem de ter “cara de médico”, nos lembra o autor.

O artigo seguinte da coletânea nos lembra que nem sempre o racismo ocorre de maneira direta. Muitas vezes, seus efeitos aparecem como uma “presença ausente”, ideia sugerida por M’Charek et al. ^6^ e utilizada pelo autor como referencial de análise. Partindo da instigante pergunta - É preciso racializar o cuidado? -, ele reflete sobre a importância de se considerar as especificidades da saúde da população negra quando se planeja a organização do cuidado.

Neste momento, o livro nos conduz a um debate ainda inacabado, que tomou corpo no contexto de implementação da Política Nacional de Saúde Integral da População Negra e que coloca de um lado os que defendem a racialização do cuidado como uma proposta positiva para a efetivação da universalização e da equidade e, de outro, as forças contrárias, que não reconhecem o racismo como um determinante social de saúde e negativamente classificam o campo saúde da população negra como um esforço de “racialização das políticas de saúde”, atribuindo a ele o distanciamento da luta pela universalização da saúde ^7^.

A questão das especificidades em saúde segue presente nos capítulos finais do livro, nos quais são desenvolvidas discussões interessantes sobre o racismo obstétrico e práticas específicas de letramento racial voltadas a vítimas de violência obstétrica, e sobre o autocuidado de mulheres negras com base no cultivo de plantas.
